# Climate data for hygrothermal simulations of Brussels

**DOI:** 10.1016/j.dib.2022.108491

**Published:** 2022-07-26

**Authors:** Isabeau Vandemeulebroucke, Steven Caluwaerts, Nathan Van Den Bossche

**Affiliations:** a Building Physics Group, Faculty of Engineering and Architecture, Ghent University, UGent Campus UFO, Technicum T4, Sint-Pietersnieuwstraat 41, Gent 9000, Belgium; b Atmospheric Physics Group, Faculty of Science, Ghent University, UGent Campus Sterre - S 9, Krijgslaan 281, Gent 9000, Belgium; c Department Meteorological and Climatological Research, Royal Meteorological Institute of Belgium, 3 Avenue Circulaire, Brussels 1180, Belgium

**Keywords:** Heat Air Moisture (HAM) simulations, Moisture Reference Year (MRY), Building envelope, Meteorological data, Degradation mechanisms, Moisture damage, Delphin, WUFI

## Abstract

Moisture is a dominant agent in the degradation of building components. To assess degradation phenomena in building envelopes, hygrothermal simulations are performed. The hygrothermal behaviour of building envelopes depends on the outdoor climate conditions. Therefore, it is important to use climate data near the location of interest when running hygrothermal simulations.

There are no appropriate climate data for hygrothermal simulations of Belgian cities. Therefore, we created two types of climate files for Brussels, i.e. the capital of Belgium. This paper presents the climate data that are selected based on the framework developed by Vandemeulebroucke et al. The first climate file is a long-term 30-year climate file. Using this climate file is the most reliable, but computationally expensive. This climate file is recommended for academics having sufficient computational power. The second file is a Moisture Reference Year (MRY), which is one year of climate data that represents the 90th percentile of moisture stress on building envelopes. The MRY is selected based on a climate index that is critical for many applications, i.e. the free wind-driven rain load. The reference year is called a generic climate-based MRY. The latter climate file is recommended for building consultants or academics performing very large studies, as it requires less computational power at the cost of a lower level of detail.

## Specifications Table

**Table:** 

Subject	Civil and Structural Engineering
Specific subject area	hygrothermal modelling, durability, building components
Type of data	Climate files for hygrothermal simulations
How the data were acquired	The raw climate data were provided by the Royal Meteorological Institute of Belgium. Then, the data were processed with R-language to fill the gaps in the data, and compute additional variables. Following on this, either conditioning years were included (long-term 30-year climate data), or a subset of the data was made (generic climate-based moisture reference year).
Data format	Analyzed, Filtered, Processed
Description of data collection	The outdoor air temperature, relative humidity, wind direction and velocity, precipitation, cloud cover, and direct and diffuse shortwave radiation over the period 1987–2017 were collected. The downward longwave radiation was computed based on Finkenstein and Häupl [2]. The indoor conditions, i.e. temperature and relative humidity, were computed based on EN 15026 [3] and WTA 6.2 [4].
Data source location	Institution: Royal Meteorological Institute of Belgium City/Town/Region: Brussels-Capital Region Country: Belgium Latitude and longitude (and GPS coordinates, if possible) for collected samples/data: 50.8° N, 4.3° E
Data accessibility	The Royal Meteorological Institute of Belgium are creating an open access database that will give access to the raw data (work-in-progress). Direct URL to database: https://opendata.meteo.be/downloadPage.php The secondary data, i.e. the climate files for hygrothermal simulations, are available as supplementary files (DOI: 10.17632/r2xycwz8xf.1). Instructions for accessing these data: The data can be accessed on https://data.mendeley.com/datasets/r2xycwz8xf/1.
Related research article	I. Vandemeulebroucke, S. Caluwaerts, N. Van Den Bossche, Decision framework to select moisture reference years for hygrothermal simulations, Building and Environment 218 (2022). https://doi.org/10.1016/j.buildenv.2022.109080

## Value of the Data

• In literature, there are studies on generating climate files for building simulations [5], [6], [7]. Generally, these studies focus on building energy simulations to compute heating/cooling loads, energy performance. The selected climate data are often based on temperature and solar radiation. However, the climate files in this work are generated for hygrothermal simulations to study the durability and deterioration of building components. The requirements are very different, i.e. the moisture load is the most important variable.
• Today, hygrothermal simulations on buildings in Belgium are usually performed using the location of Essen, i.e. a near location in Germany, due to the lack of climate files of cities in Belgium. However, there are large regional differences in climatological conditions. Using the climate file of Essen does not result in an accurate assessment of the hygrothermal behaviour of building envelopes in Belgium. Therefore, we created climate files for Brussels, i.e. the capital of Belgium.
• A range of different people can benefit from these climate files, e.g. academics, building and/or renovation consultants, and researchers in the building industry*.*
• The climate files are ready-to-use in hygrothermal software, e.g. Delphin 6. No complicated pre-processing of climate data is required. This makes the process of simulations more accessible to building practitioners.
• These climate files have a wide range of potential uses. They can be used as outdoor climate conditions in sensitivity analyses to understand the impact of parameter variations like material properties, wall configuration, rain exposure etc. Further, they can be used to study the durability of new materials, e.g. bio-based insulation materials, or to evaluate renovation strategies, such as the application of interior insulation.

## 1 Data Description

The data consists of two sets of climate data for hygrothermal simulations in Brussels. The two sets of climate data are produced based on the decision framework by Vandemeulebroucke et al. [1]. The decision framework offers different approaches to select climate data for hygrothermal simulations with respect to the available computational resources and the desired level of detail.

The first set of climate data is a long-term 30-year climate file for HAM tools. This climate file can be used to perform a ‘full response-based simulation’. This means that the complete 30-year (or 31-year) climate is simulated. Then, the result of the simulation is a complete 30-year response of the building envelope (e.g. a temperature and relative humidity profile for 30 years of hourly simulation results). This approach requires a high computational power, but provides the most reliable results.

The second set of climate data is a Moisture Reference Year (MRY), which is one year of climate data with a 10-year return period in terms of moisture stress on building envelopes on a specific location [8], [9], [10]. The MRY is often selected based on a climate index, e.g. the Moisture Index [8], which depends only on climate variables. The chosen climate index is known to have a correlation with the hygrothermal performance of building envelopes at the indicated location. This set of climate data is called a ‘generic climate-based MRY’.

To generate these two sets of climate data for hygrothermal simulations, raw climate data are required. In this dataset, the raw climate data are based on the hourly measurements of meteorological variables in Brussels between 1987 and 2017.

Further, the long-term climate file contains the entire period 1987–2017. The generic climate-based MRY, on the other hand, only contains one year of climate data, which is selected using a climate index. In this particular case, the chosen climate index is the free wind-driven rain load. The resulting MRY is the year October 1st 1987–September 30th, i.e. having a 10 year return period in terms of free wind-driven rain load. Note that the MRY starts on October 1st, since this improves the results compared to the long-term simulation [9,10].

Hygrothermal simulations require a conditioning period, i.e. a spin-up period of the simulation. The conditioning period is required to eliminate the effect of the initial conditions of the building components. Note that the conditioning years are not considered in the analysis of the simulation results. For the long-term climate file, the conditioning period is equal to the first 4 years of the climate data, i.e. January 1st 1987 to December 31st 1990. Then, the evaluation period consists of the entire 31-year climate dataset, i.e. January 1st 1987 to December 31st 2017. In other words, the years 1987–1990 are simulated twice, followed by 1991–2017. The evaluation period can be analysed in its entirety with evaluation years starting on January 1st. In some studies, however, the evaluation period starts on October 1st. In that case, the analysis is performed on the period October 1st 1987–September 30th 2017, i.e. a 30-year period, as is the case in the study by Vandemeulebroucke et al. [1].

Secondly, the generic climate-based MRY consists of 1 year of climate data, i.e. October 1st 1987–September 30th 1988. The number of conditioning years can be chosen freely by repeating the MRY several times, e.g. 5 times. The last year is the evaluation period. For convenience, the order of the year is modified so the climate file starts on January 1st (January 1st–September 30th 1988, followed by October 1st–December 31st 1987). Since the MRY is repeated a number of times to condition the wall assembly, this does not influence the results.

The data are organized as follows. Each sets of climate data consists of 3 files: (i) outdoor climate conditions, (ii) indoor air temperature, and (iii) relative humidity of the indoor air. Considering the outdoor climate conditions, the description of the columns in the files are listed in Table 1. The file also provides information on the latitude, longitude, height above mean sea level, time zone, and time step. The file format is *.wac. Further, for the indoor air temperature and relative humidity, the columns are organized in the following way: the day of the year with 0 equal to January 1st (Column 1), the time of the day in [hours:minutes:seconds] (Column 2), and the temperature or relative humidity in [°C] or [%], respectively. The file format of the indoor climate conditions is *.ccd.

**Table 1:** Description column names in files with outdoor climate conditions

Column name	Description
TIME	Time stamp in [year-month-day hour:minutes]
TA	Air temperature [°C]
HREL	Relative humidity [0–1]
ISDH	Direct shortwave radiation [W/m²]
ISD	Diffuse shortwave radiation [W/m²]
RN	Precipitation [l/m²h]
WD	Wind direction [°, with north=0° and east=90°]
WS	Wind velocity [m/s]
WV	Wind velocity [m/s]
ILAH	Downward longwave radiation [W/m²]

Note that the meteorological data are provided by the Royal Meteorological Institute (RMI) of Belgium, whereas the framework to select the climate is developed by Vandemeulebroucke et al. [1]. Therefore, the RMI, this paper, and Vandemeulebroucke et al. [1] should be cited in each publication in which this data is used.

Furthermore, other MRYs based on existing methods can be extracted from the long-term climate file. For other MRYs, the selected years for Brussels (1987–2017) are indicated in Table A in Appendix A.

## 2 Experimental Design, Materials and Methods

### 2.1 Raw Data

The raw climate variables are measured in Uccle, which is the location of the headquarters of the Royal Meteorological institute of Belgium. Uccle is a municipality located south of the city centre of Brussels, and is part of the Brussels-Capital Region. The raw data covers the period 1987–2017, and consists of hourly values of the following variables: outdoor air temperature, relative humidity, wind direction and velocity, precipitation, cloud cover, and direct and diffuse shortwave radiation.

### 2.2 Gaps

The gaps in the data are filled with measurement data at the National Airport in Zaventem, which is located north-east of Brussels’ city centre. After the filling the gaps with these data, the size of the remaining gaps is maximum 8 h for temperature and relative humidity, 11 h for wind velocity, 12 h for wind direction, and 26 h for cloud cover. These remaining gaps are filled by applying linear interpolation. For precipitation, direct and diffuse shortwave radiation, there are no gaps after the correction with the data of the National Airport.

### 2.3 Missing Variables

The downward longwave radiation is not available over the entire period, and is therefore calculated using the air temperature, relative humidity, and cloud cover. The methodology by Finkenstein and Häupl [2] is applied, as they studied procedures to compute the downward longwave radiation for hygrothermal simulations. The method uses the Stefan-Boltzmann law:

$$q=\varepsilon \sigma T^{4}$$

with the downward longwave radiation q [W/m²], emission coefficient ε, Stefan-Boltzmann coefficient σ and air temperature T [K].

The emission coefficient depends on the cloud cover. For a clear sky, the emission coefficient $\varepsilon_{\text{clear}}$ is based on the Brutsaert equation, as it was found the best overall equation by Finkenstein and Häupl [2]. The Konzelmann equation is selected to calculate the emission coefficient for all skies $\varepsilon_{\text{all}}$, again based on Finkenstein and Häupl [2].

$$\varepsilon_{\text{clear}}=1.24\cdot {\left(\frac{p_{0}}{T_{0}}\right)}^{\frac{1}{7}}$$

$$\varepsilon_{\text{all}}=\varepsilon_{\text{clear}}\left(1-n^{4}\right)+0.952\cdot n^{4}$$

with the vapour pressure at sea-level $p_{0}$ [hPa], temperature near the surface $T_{0}$ [K], and the cloud index n [-].

### 2.4 Indoor Climate Conditions

The indoor climate conditions are calculated according to the DIN EN 15026/WTA adaptive indoor climate model [3,4]. The indoor climate model for ‘increased moisture load (plus 5%)’ is applied according to WTA 6.2 [4]. The indoor temperature and relative humidity range between 20 and 25°C and 35 and 65%, respectively, depending on the outdoor air temperature.

## Ethics Statements

The work complies with the ethical guidelines of the ‘Data in Brief’ journal. Further, the work did not involve human subjects, animal experiments, or data collected from social media platforms.

## CRediT authorship contribution statement

**Isabeau Vandemeulebroucke:** Methodology, Data curation, Writing – original draft. **Steven Caluwaerts:** Resources, Supervision, Writing – review & editing. **Nathan Van Den Bossche:** Methodology, Supervision, Writing – review & editing.

## Declaration of Competing Interest

The authors declare that they have no known competing financial interests or personal relationships that could have appeared to influence the work reported in this paper.

## Data Availability

Climate files for hygrothermal simulations of Brussels (Original data) (Mendeley Data).

## Appendix Supplementary materials

Image, application 1
