# Supplementary material for: Climate data for hygrothermal simulations of Brussels
Source: Data Brief. 2022 Jul 26;44:108491. doi: 10.1016/j.dib.2022.108491 (PMC9399475; doi:10.1016/j.dib.2022.108491)
Supplement: Supplementary file 1 [file mmc1.docx]

**Appendix A**

Table A Existing methods to select Moisture Reference Years (MRY) result in a range of different years. Some years follow the calendar year, whereas others start on October 1st or July 1st. (Modified from Vandemeulebroucke et al. [1]).

| **Selected year for Brussels (1987-2017)** | **Description** | **Publication** |
| --- | --- | --- |
| 1999 | Construction-dependent method based on the integral of the moisture content of the wall | C. Rode, “Reference years for moisture calculations, Denmark. Report T2-DK-93/02, IEA Annex 24, HAMTIE.,” 1993. |
| Jul 1, 2007 -Jun 30, 2008 | Saturation deficit | T. Kalamees and J. Vinha, “Estonian Climate Analysis for Selecting Moisture Reference Years for Hygrothermal Calculations,” *J. Build. Phys.*, vol. 27, no. 3, pp. 199–220, 2004. |
| Jul 1, 2002 -Jun 30, 2003 | Mould index based on air temperature and relative humidity | T. Kalamees and J. Vinha, “Estonian Climate Analysis for Selecting Moisture Reference Years for Hygrothermal Calculations,” *J. Build. Phys.*, vol. 27, no. 3, pp. 199–220, 2004. |
| 1988 | Moisture index | S. Cornick, R. Djebbar, and W. Alan Dalgliesh, “Selecting moisture reference years using a Moisture Index approach,” *Build. Environ.*, vol. 38, no. 12, pp. 1367–1379, 2003. |
| Oct 1, 1987 -Sep 30, 1988 | Free wind-driven rain load | R. E. Lacy, “Driving-rain maps and the onslaught of rain on buildings,” 1965. |
| Oct 1, 2001 - Sep 30, 2002 | Wind-driven rain from the critical orientation | CEN, “EN ISO 15927-3: Hygrothermal performance of buildings - Calculation and presentation of climatic data - Part 3: Calculation of a driving rain index for vertical surfaces from hourly wind and rain data.” 2009. |
| 2002 | Severity Index | M. Salonvaara, K. Sedlbauer, A. Holm, and M. Pazera, “Effect of Selected Weather Year for Hygrothermal Analyses,” in *Proceedings of the Thermal Performance of the Exterior Envelopes of Whole Buildings XI International Conference*, 2010, pp. 1–12. |
| Oct 1, 1987 - Sep 30, 1988 | Climatic Index | X. Zhou, D. Derome, and J. Carmeliet, “Robust moisture reference year methodology for hygrothermal simulations,” *Build. Environ.*, vol. 110, pp. 23–35, 2016. |
| Oct 1, 1987 - Sep 30, 1988;  Oct 1, 2000 - Sep 30, 2001;  Oct 1, 1997 - Sep 30, 1998 | Climatic Index (3th, 4th and 5th year in decreasing order), performing hygrothermal simulations and selecting most critical year based on RHT index | X. Zhou, D. Derome, and J. Carmeliet, “Robust moisture reference year methodology for hygrothermal simulations,” *Build. Environ.*, vol. 110, pp. 23–35, 2016. |
| synthetized | - Extreme high year of precipitation - Typical design year of precipitation - Extreme high year of air temperature - Extreme low year of air temperature - Typical design year of air temperature - Typical design year of precipitation (conditioning period) followed by extreme high year - Typical design year of air temperature (conditioning period) followed by extreme high year - Typical design year of air temperature (conditioning period) followed by extreme low year | V. M. Nik, “Making energy simulation easier for future climate - Synthesizing typical and extreme weather data sets out of regional climate models (RCMs),” *Appl. Energy*, vol. 177, pp. 204–226, 2016.  V. M. Nik, “Application of typical and extreme weather data sets in the hygrothermal simulation of building components for future climate – A case study for a wooden frame wall,” *Energy Build.*, vol. 154, pp. 30–45, 2017. |
| Oct 1, 2010 - Sep 30, 2011 | Freeze-thaw cycles based on air temperature | C. M. Grossi, P. Brimblecombe, and I. Harris, “Predicting long term freeze-thaw risks on Europe built heritage and archaeological sites in a changing climate,” *Sci. Total Environ.*, vol. 377, pp. 273–281, 2007. |
| Oct 1, 1998 - Sep 30, 1999 | Frost decay exposure index | K. R. Lisø, T. Kvande, H. O. Hygen, J. V. Thue, and K. Harstveit, “A frost decay exposure index for porous, mineral building materials,” *Build. Environ.*, vol. 42, no. 10, pp. 3547–3555, 2007. |
| No year found | Wet-frost index | P. Brimblecombe, C. M. Grossi, and I. Harris, “Climate change critical to cultural heritage,” in *Survival and Sustainability. Environmental Earth Sciences.*, H. Gökçekus, U. Türker, and J. LaMoreaux, Eds. Berlin, Heidelberg: Springer, 2011. |
